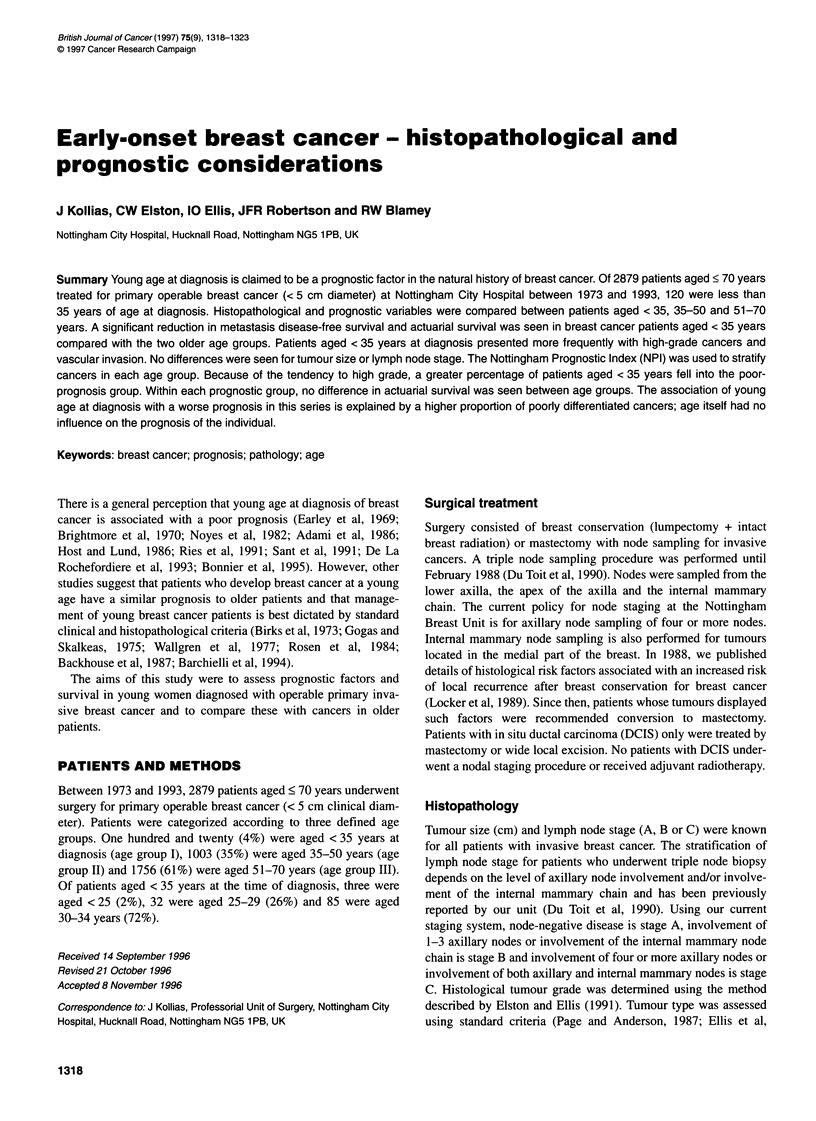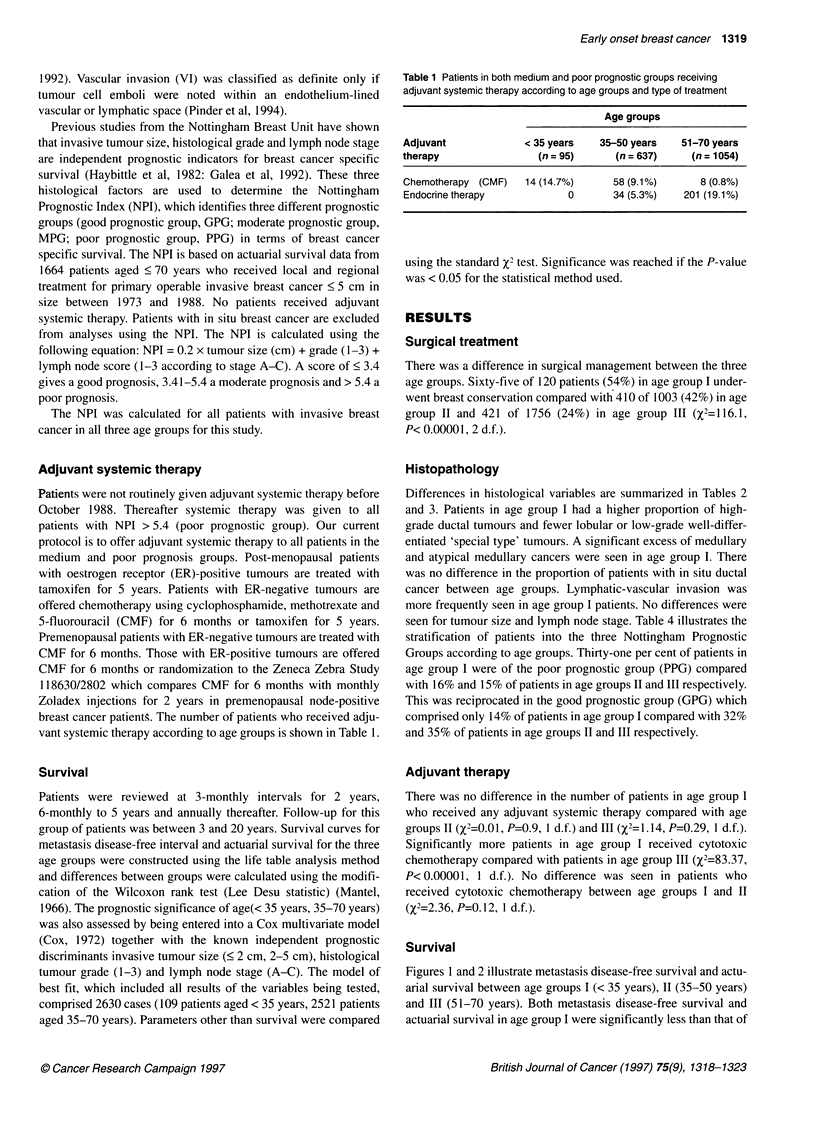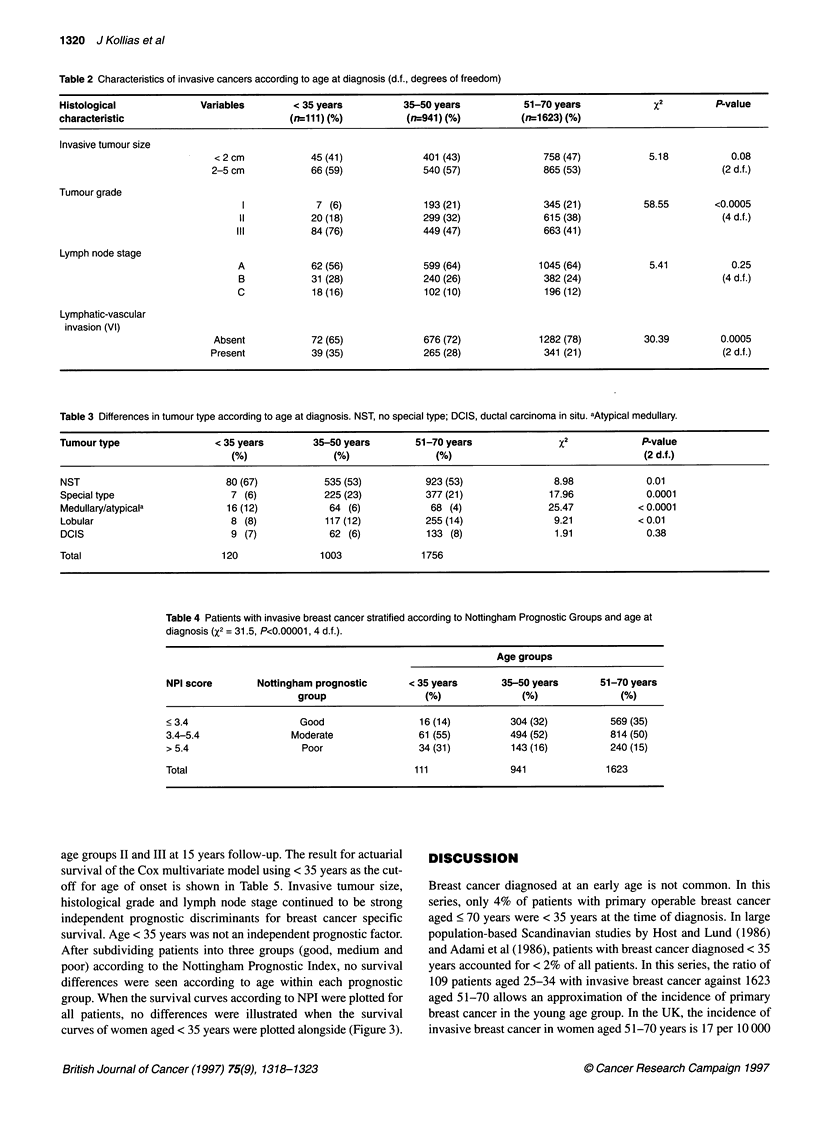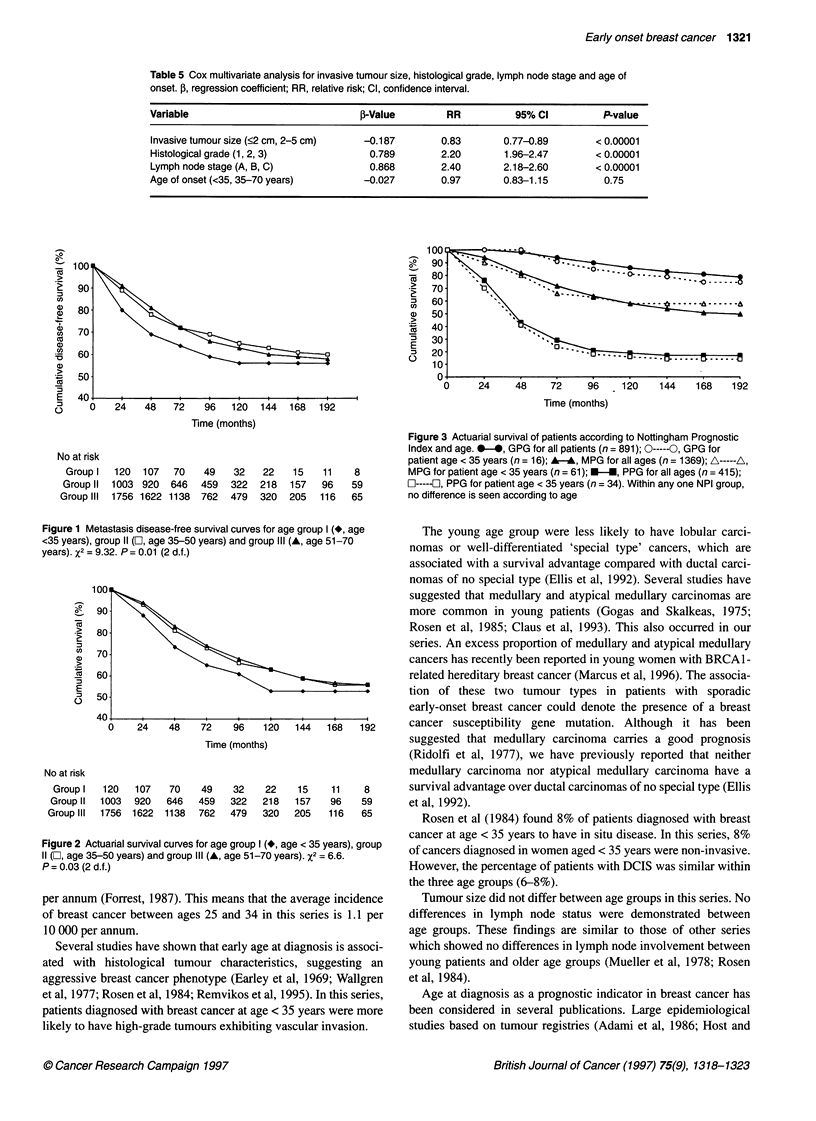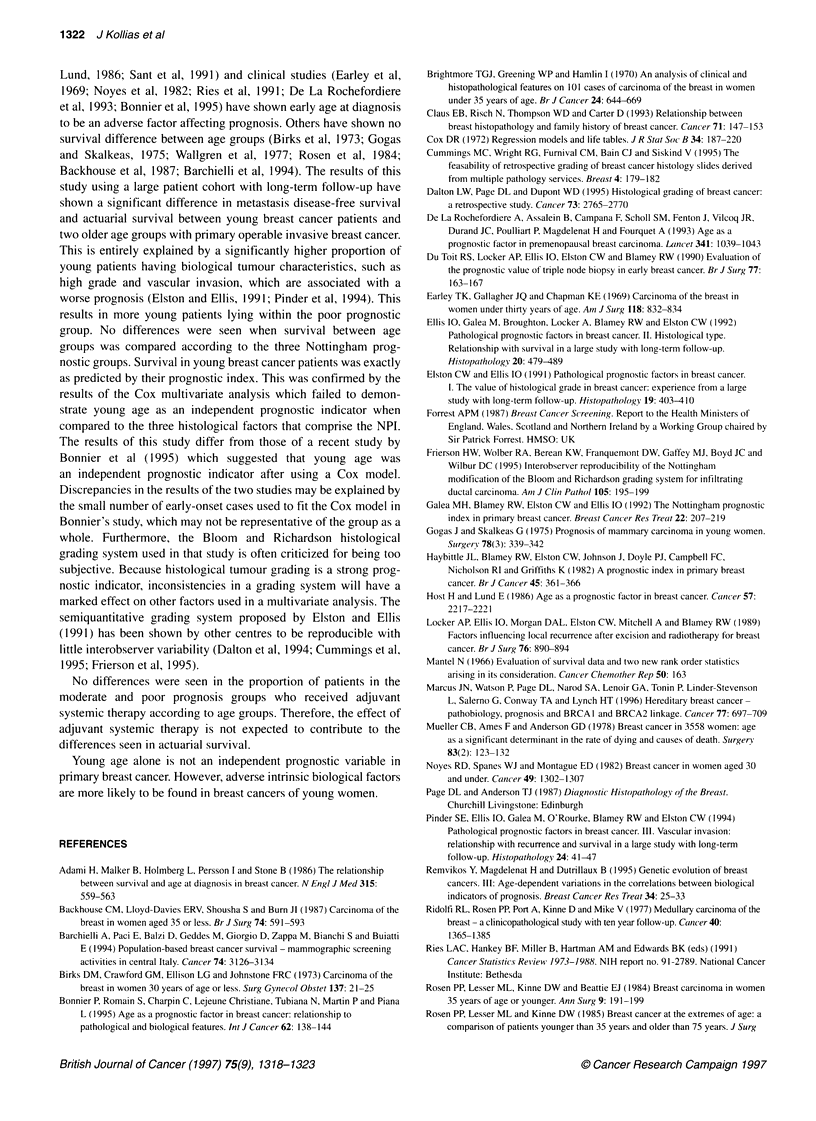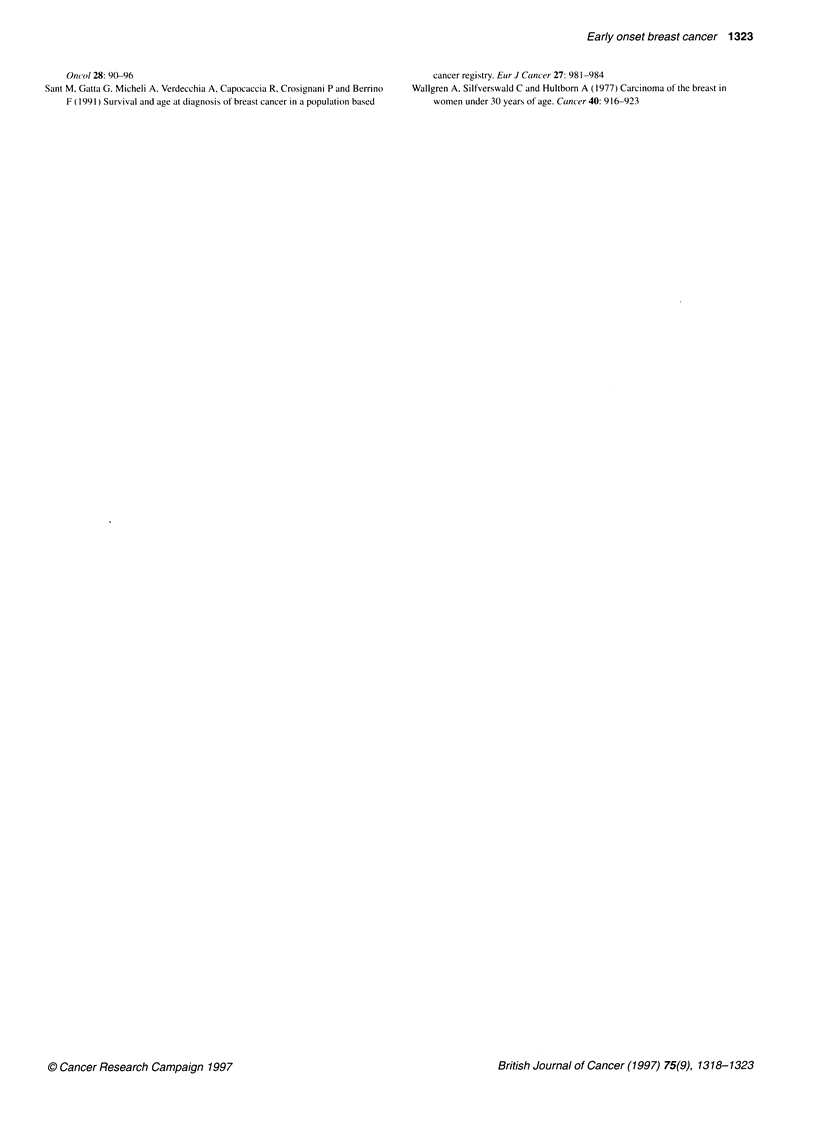# Early-onset breast cancer--histopathological and prognostic considerations.

**DOI:** 10.1038/bjc.1997.223

**Published:** 1997

**Authors:** J. Kollias, C. W. Elston, I. O. Ellis, J. F. Robertson, R. W. Blamey

**Affiliations:** Nottingham City Hospital, UK.

## Abstract

Young age at diagnosis is claimed to be a prognostic factor in the natural history of breast cancer. Of 2879 patients aged < 70 years treated for primary operable breast cancer (< 5 cm diameter) at Nottingham City Hospital between 1973 and 1993, 120 were less than 35 years of age at diagnosis. Histopathological and prognostic variables were compared between patients aged < 35, 35-50 and 51-70 years. A significant reduction in metastasis disease-free survival and actuarial survival was seen in breast cancer patients aged < 35 years compared with the two older age groups. Patients aged < 35 years at diagnosis presented more frequently with high-grade cancers and vascular invasion. No differences were seen for tumour size or lymph node stage. The Nottingham Prognostic Index (NPI) was used to stratify cancers in each age group. Because of the tendency to high grade, a greater percentage of patients aged < 35 years fell into the poor-prognosis group. Within each prognostic group, no difference in actuarial survival was seen between age groups. The association of young age at diagnosis with a worse prognosis in this series is explained by a higher proportion of poorly differentiated cancers; age itself had no influence on the prognosis of the individual.


British Journal of Cancer (1997) 75(9), 1318–1323
© 1997 Cancer Research Campaign

### Early-onset breast cancer – histopathological and prognostic considerations

**J Kollias, CW Elston, IO Ellis, JFR Robertson and RW Blamey**

Nottingham City Hospital, Hucknall Road, Nottingham NG5 1PB, UK

**Summary** Young age at diagnosis is claimed to be a prognostic factor in the natural history of breast cancer. Of 2879 patients aged ≤ 70 years treated for primary operable breast cancer (< 5 cm diameter) at Nottingham City Hospital between 1973 and 1993, 120 were less than 35 years of age at diagnosis. Histopathological and prognostic variables were compared between patients aged < 35, 35–50 and 51–70 years. A significant reduction in metastasis disease-free survival and actuarial survival was seen in breast cancer patients aged < 35 years compared with the two older age groups. Patients aged < 35 years at diagnosis presented more frequently with high-grade cancers and vascular invasion. No differences were seen for tumour size or lymph node stage. The Nottingham Prognostic Index (NPI) was used to stratify cancers in each age group. Because of the tendency to high grade, a greater percentage of patients aged < 35 years fell into the poor-prognosis group. Within each prognostic group, no difference in actuarial survival was seen between age groups. The association of young age at diagnosis with a worse prognosis in this series is explained by a higher proportion of poorly differentiated cancers; age itself had no influence on the prognosis of the individual.

**Keywords**: breast cancer; prognosis; pathology; age

There is a general perception that young age at diagnosis of breast cancer is associated with a poor prognosis (Earley et al, 1969; Brightmore et al, 1970; Noyes et al, 1982; Adami et al, 1986; Host and Lund, 1986; Ries et al, 1991; Sant et al, 1991; De La Rochefordiere et al, 1993; Bonnier et al, 1995). However, other studies suggest that patients who develop breast cancer at a young age have a similar prognosis to older patients and that management of young breast cancer patients is best dictated by standard clinical and histopathological criteria (Birks et al, 1973; Gogas and Skalkeas, 1975; Wallgren et al, 1977; Rosen et al, 1984; Backhouse et al, 1987; Barchielli et al, 1994).

The aims of this study were to assess prognostic factors and survival in young women diagnosed with operable primary invasive breast cancer and to compare these with cancers in older patients.

### PATIENTS AND METHODS

Between 1973 and 1993, 2879 patients aged ≤ 70 years underwent surgery for primary operable breast cancer (< 5 cm clinical diameter). Patients were categorized according to three defined age groups. One hundred and twenty (4%) were aged < 35 years at diagnosis (age group I), 1003 (35%) were aged 35–50 years (age group II) and 1756 (61%) were aged 51–70 years (age group III). Of patients aged < 35 years at the time of diagnosis, three were aged < 25 (2%), 32 were aged 25–29 (26%) and 85 were aged 30–34 years (72%).

*Received 14 September 1996*
*Revised 21 October 1996*
*Accepted 8 November 1996*

*Correspondence to:* J Kollias, Professorial Unit of Surgery, Nottingham City Hospital, Hucknall Road, Nottingham NG5 1PB, UK

### Surgical treatment

Surgery consisted of breast conservation (lumpectomy + intact breast radiation) or mastectomy with node sampling for invasive cancers. A triple node sampling procedure was performed until February 1988 (Du Toit et al, 1990). Nodes were sampled from the lower axilla, the apex of the axilla and the internal mammary chain. The current policy for node staging at the Nottingham Breast Unit is for axillary node sampling of four or more nodes. Internal mammary node sampling is also performed for tumours located in the medial part of the breast. In 1988, we published details of histological risk factors associated with an increased risk of local recurrence after breast conservation for breast cancer (Locker et al, 1989). Since then, patients whose tumours displayed such factors were recommended conversion to mastectomy. Patients with in situ ductal carcinoma (DCIS) only were treated by mastectomy or wide local excision. No patients with DCIS underwent a nodal staging procedure or received adjuvant radiotherapy.

### Histopathology

Tumour size (cm) and lymph node stage (A, B or C) were known for all patients with invasive breast cancer. The stratification of lymph node stage for patients who underwent triple node biopsy depends on the level of axillary node involvement and/or involvement of the internal mammary chain and has been previously reported by our unit (Du Toit et al, 1990). Using our current staging system, node-negative disease is stage A, involvement of 1–3 axillary nodes or involvement of the internal mammary node chain is stage B and involvement of four or more axillary nodes or involvement of both axillary and internal mammary nodes is stage C. Histological tumour grade was determined using the method described by Elston and Ellis (1991). Tumour type was assessed using standard criteria (Page and Anderson, 1987; Ellis et al,

1992). Vascular invasion (VI) was classified as definite only if tumour cell emboli were noted within an endothelium-lined vascular or lymphatic space (Pinder et al, 1994).

Previous studies from the Nottingham Breast Unit have shown that invasive tumour size, histological grade and lymph node stage are independent prognostic indicators for breast cancer specific survival (Haybittle et al, 1982: Galea et al, 1992). These three histological factors are used to determine the Nottingham Prognostic Index (NPI), which identifies three different prognostic groups (good prognostic group, GPG; moderate prognostic group, MPG; poor prognostic group, PPG) in terms of breast cancer specific survival. The NPI is based on actuarial survival data from 1664 patients aged $\leq 70$ years who received local and regional treatment for primary operable invasive breast cancer $\leq 5$ cm in size between 1973 and 1988. No patients received adjuvant systemic therapy. Patients with in situ breast cancer are excluded from analyses using the NPI. The NPI is calculated using the following equation: NPI = 0.2 × tumour size (cm) + grade (1–3) + lymph node score (1–3 according to stage A–C). A score of $\leq 3.4$ gives a good prognosis, 3.41–5.4 a moderate prognosis and > 5.4 a poor prognosis.

The NPI was calculated for all patients with invasive breast cancer in all three age groups for this study.

### Adjuvant systemic therapy

Patients were not routinely given adjuvant systemic therapy before October 1988. Thereafter systemic therapy was given to all patients with NPI > 5.4 (poor prognostic group). Our current protocol is to offer adjuvant systemic therapy to all patients in the medium and poor prognosis groups. Post-menopausal patients with oestrogen receptor (ER)-positive tumours are treated with tamoxifen for 5 years. Patients with ER-negative tumours are offered chemotherapy using cyclophosphamide, methotrexate and 5-fluorouracil (CMF) for 6 months or tamoxifen for 5 years. Premenopausal patients with ER-negative tumours are treated with CMF for 6 months. Those with ER-positive tumours are offered CMF for 6 months or randomization to the Zeneca Zebra Study 118630/2802 which compares CMF for 6 months with monthly Zoladex injections for 2 years in premenopausal node-positive breast cancer patients. The number of patients who received adjuvant systemic therapy according to age groups is shown in Table 1.

### Survival

Patients were reviewed at 3-monthly intervals for 2 years, 6-monthly to 5 years and annually thereafter. Follow-up for this group of patients was between 3 and 20 years. Survival curves for metastasis disease-free interval and actuarial survival for the three age groups were constructed using the life table analysis method and differences between groups were calculated using the modification of the Wilcoxon rank test (Lee Desu statistic) (Mantel, 1966). The prognostic significance of age(< 35 years, 35–70 years) was also assessed by being entered into a Cox multivariate model (Cox, 1972) together with the known independent prognostic discriminants invasive tumour size ($\leq 2$ cm, 2–5 cm), histological tumour grade (1–3) and lymph node stage (A–C). The model of best fit, which included all results of the variables being tested, comprised 2630 cases (109 patients aged < 35 years, 2521 patients aged 35–70 years). Parameters other than survival were compared

**Table 1** Patients in both medium and poor prognostic groups receiving adjuvant systemic therapy according to age groups and type of treatment

| | Age groups | | |
|---|---|---|---|
| **Adjuvant therapy** | **< 35 years (*n* = 95)** | **35–50 years (*n* = 637)** | **51–70 years (*n* = 1054)** |
| Chemotherapy (CMF) | 14 (14.7%) | 58 (9.1%) | 8 (0.8%) |
| Endocrine therapy | 0 | 34 (5.3%) | 201 (19.1%) |

using the standard $\chi^2$ test. Significance was reached if the *P*-value was < 0.05 for the statistical method used.

### RESULTS

### Surgical treatment

There was a difference in surgical management between the three age groups. Sixty-five of 120 patients (54%) in age group I underwent breast conservation compared with 410 of 1003 (42%) in age group II and 421 of 1756 (24%) in age group III ($\chi^2$=116.1, $P$< 0.00001, 2 d.f.).

### Histopathology

Differences in histological variables are summarized in Tables 2 and 3. Patients in age group I had a higher proportion of high-grade ductal tumours and fewer lobular or low-grade well-differentiated 'special type' tumours. A significant excess of medullary and atypical medullary cancers were seen in age group I. There was no difference in the proportion of patients with in situ ductal cancer between age groups. Lymphatic-vascular invasion was more frequently seen in age group I patients. No differences were seen for tumour size and lymph node stage. Table 4 illustrates the stratification of patients into the three Nottingham Prognostic Groups according to age groups. Thirty-one per cent of patients in age group I were of the poor prognostic group (PPG) compared with 16% and 15% of patients in age groups II and III respectively. This was reciprocated in the good prognostic group (GPG) which comprised only 14% of patients in age group I compared with 32% and 35% of patients in age groups II and III respectively.

### Adjuvant therapy

There was no difference in the number of patients in age group I who received any adjuvant systemic therapy compared with age groups II ($\chi^2$=0.01, $P$=0.9, 1 d.f.) and III ($\chi^2$=1.14, $P$=0.29, 1 d.f.). Significantly more patients in age group I received cytotoxic chemotherapy compared with patients in age group III ($\chi^2$=83.37, $P$< 0.00001, 1 d.f.). No difference was seen in patients who received cytotoxic chemotherapy between age groups I and II ($\chi^2$=2.36, $P$=0.12, 1 d.f.).

### Survival

Figures 1 and 2 illustrate metastasis disease-free survival and actuarial survival between age groups I (< 35 years), II (35–50 years) and III (51–70 years). Both metastasis disease-free survival and actuarial survival in age group I were significantly less than that of

© Cancer Research Campaign 1997

**Table 2** Characteristics of invasive cancers according to age at diagnosis (d.f., degrees of freedom)

| Histological characteristic | Variables | < 35 years (*n*=111) (%) | 35–50 years (*n*=941) (%) | 51–70 years (*n*=1623) (%) | $\chi^2$ | *P*-value |
|---|---|---|---|---|---|---|
| Invasive tumour size | | | | | | |
| | < 2 cm | 45 (41) | 401 (43) | 758 (47) | 5.18 | 0.08 |
| | 2–5 cm | 66 (59) | 540 (57) | 865 (53) | | (2 d.f.) |
| Tumour grade | | | | | | |
| | I | 7 (6) | 193 (21) | 345 (21) | 58.55 | <0.0005 |
| | II | 20 (18) | 299 (32) | 615 (38) | | (4 d.f.) |
| | III | 84 (76) | 449 (47) | 663 (41) | | |
| Lymph node stage | | | | | | |
| | A | 62 (56) | 599 (64) | 1045 (64) | 5.41 | 0.25 |
| | B | 31 (28) | 240 (26) | 382 (24) | | (4 d.f.) |
| | C | 18 (16) | 102 (10) | 196 (12) | | |
| Lymphatic-vascular invasion (VI) | | | | | | |
| | Absent | 72 (65) | 676 (72) | 1282 (78) | 30.39 | 0.0005 |
| | Present | 39 (35) | 265 (28) | 341 (21) | | (2 d.f.) |

**Table 3** Differences in tumour type according to age at diagnosis. NST, no special type; DCIS, ductal carcinoma in situ. [a]Atypical medullary.

| Tumour type | < 35 years (%) | 35–50 years (%) | 51–70 years (%) | $\chi^2$ | *P*-value (2 d.f.) |
|---|---|---|---|---|---|
| NST | 80 (67) | 535 (53) | 923 (53) | 8.98 | 0.01 |
| Special type | 7 (6) | 225 (23) | 377 (21) | 17.96 | 0.0001 |
| Medullary/atypical[a] | 16 (12) | 64 (6) | 68 (4) | 25.47 | < 0.0001 |
| Lobular | 8 (8) | 117 (12) | 255 (14) | 9.21 | < 0.01 |
| DCIS | 9 (7) | 62 (6) | 133 (8) | 1.91 | 0.38 |
| Total | 120 | 1003 | 1756 | | |

**Table 4** Patients with invasive breast cancer stratified according to Nottingham Prognostic Groups and age at diagnosis ($\chi^2$ = 31.5, *P*<0.00001, 4 d.f.).

| | | Age groups | | |
|---|---|---|---|---|
| NPI score | Nottingham prognostic group | < 35 years (%) | 35–50 years (%) | 51–70 years (%) |
| ≤ 3.4 | Good | 16 (14) | 304 (32) | 569 (35) |
| 3.4–5.4 | Moderate | 61 (55) | 494 (52) | 814 (50) |
| > 5.4 | Poor | 34 (31) | 143 (16) | 240 (15) |
| Total | | 111 | 941 | 1623 |

age groups II and III at 15 years follow-up. The result for actuarial survival of the Cox multivariate model using < 35 years as the cut-off for age of onset is shown in Table 5. Invasive tumour size, histological grade and lymph node stage continued to be strong independent prognostic discriminants for breast cancer specific survival. Age < 35 years was not an independent prognostic factor. After subdividing patients into three groups (good, medium and poor) according to the Nottingham Prognostic Index, no survival differences were seen according to age within each prognostic group. When the survival curves according to NPI were plotted for all patients, no differences were illustrated when the survival curves of women aged < 35 years were plotted alongside (Figure 3).

### DISCUSSION

Breast cancer diagnosed at an early age is not common. In this series, only 4% of patients with primary operable breast cancer aged ≤ 70 years were < 35 years at the time of diagnosis. In large population-based Scandinavian studies by Host and Lund (1986) and Adami et al (1986), patients with breast cancer diagnosed < 35 years accounted for < 2% of all patients. In this series, the ratio of 109 patients aged 25–34 with invasive breast cancer against 1623 aged 51–70 allows an approximation of the incidence of primary breast cancer in the young age group. In the UK, the incidence of invasive breast cancer in women aged 51–70 years is 17 per 10 000

 © Cancer Research Campaign 1997

**Table 5** Cox multivariate analysis for invasive tumour size, histological grade, lymph node stage and age of onset. β, regression coefficient; RR, relative risk; CI, confidence interval.

| Variable | β-Value | RR | 95% CI | *P*-value |
|---|---|---|---|---|
| Invasive tumour size (≤2 cm, 2–5 cm) | –0.187 | 0.83 | 0.77–0.89 | < 0.00001 |
| Histological grade (1, 2, 3) | 0.789 | 2.20 | 1.96–2.47 | < 0.00001 |
| Lymph node stage (A, B, C) | 0.868 | 2.40 | 2.18–2.60 | < 0.00001 |
| Age of onset (<35, 35–70 years) | –0.027 | 0.97 | 0.83–1.15 | 0.75 |

No at risk

| | | | | | | | | | |
|---|---|---|---|---|---|---|---|---|---|
| Group I | 120 | 107 | 70 | 49 | 32 | 22 | 15 | 11 | 8 |
| Group II | 1003 | 920 | 646 | 459 | 322 | 218 | 157 | 96 | 59 |
| Group III | 1756 | 1622 | 1138 | 762 | 479 | 320 | 205 | 116 | 65 |

**Figure 1** Metastasis disease-free survival curves for age group I (♦, age <35 years), group II (□, age 35–50 years) and group III (▲, age 51–70 years). $\chi^2 = 9.32$. $P = 0.01$ (2 d.f.)

No at risk

| | | | | | | | | | |
|---|---|---|---|---|---|---|---|---|---|
| Group I | 120 | 107 | 70 | 49 | 32 | 22 | 15 | 11 | 8 |
| Group II | 1003 | 920 | 646 | 459 | 322 | 218 | 157 | 96 | 59 |
| Group III | 1756 | 1622 | 1138 | 762 | 479 | 320 | 205 | 116 | 65 |

**Figure 2** Actuarial survival curves for age group I (♦, age < 35 years), group II (□, age 35–50 years) and group III (▲, age 51–70 years). $\chi^2 = 6.6$. $P = 0.03$ (2 d.f.)

per annum (Forrest, 1987). This means that the average incidence of breast cancer between ages 25 and 34 in this series is 1.1 per 10 000 per annum.

Several studies have shown that early age at diagnosis is associated with histological tumour characteristics, suggesting an aggressive breast cancer phenotype (Earley et al, 1969; Wallgren et al, 1977; Rosen et al, 1984; Remvikos et al, 1995). In this series, patients diagnosed with breast cancer at age < 35 years were more likely to have high-grade tumours exhibiting vascular invasion.

**Figure 3** Actuarial survival of patients according to Nottingham Prognostic Index and age. ●—●, GPG for all patients ($n = 891$); ○-----○, GPG for patient age < 35 years ($n = 16$); ▲—▲, MPG for all ages ($n = 1369$); △-----△, MPG for patient age < 35 years ($n = 61$); ■—■, PPG for all ages ($n = 415$); □-----□, PPG for patient age < 35 years ($n = 34$). Within any one NPI group, no difference is seen according to age

The young age group were less likely to have lobular carcinomas or well-differentiated 'special type' cancers, which are associated with a survival advantage compared with ductal carcinomas of no special type (Ellis et al, 1992). Several studies have suggested that medullary and atypical medullary carcinomas are more common in young patients (Gogas and Skalkeas, 1975; Rosen et al, 1985; Claus et al, 1993). This also occurred in our series. An excess proportion of medullary and atypical medullary cancers has recently been reported in young women with BRCA1-related hereditary breast cancer (Marcus et al, 1996). The association of these two tumour types in patients with sporadic early-onset breast cancer could denote the presence of a breast cancer susceptibility gene mutation. Although it has been suggested that medullary carcinoma carries a good prognosis (Ridolfi et al, 1977), we have previously reported that neither medullary carcinoma nor atypical medullary carcinoma have a survival advantage over ductal carcinomas of no special type (Ellis et al, 1992).

Rosen et al (1984) found 8% of patients diagnosed with breast cancer at age < 35 years to have in situ disease. In this series, 8% of cancers diagnosed in women aged < 35 years were non-invasive. However, the percentage of patients with DCIS was similar within the three age groups (6–8%).

Tumour size did not differ between age groups in this series. No differences in lymph node status were demonstrated between age groups. These findings are similar to those of other series which showed no differences in lymph node involvement between young patients and older age groups (Mueller et al, 1978; Rosen et al, 1984).

Age at diagnosis as a prognostic indicator in breast cancer has been considered in several publications. Large epidemiological studies based on tumour registries (Adami et al, 1986; Host and

© Cancer Research Campaign 1997

Lund, 1986; Sant et al, 1991) and clinical studies (Earley et al, 1969; Noyes et al, 1982; Ries et al, 1991; De La Rochefordiere et al, 1993; Bonnier et al, 1995) have shown early age at diagnosis to be an adverse factor affecting prognosis. Others have shown no survival difference between age groups (Birks et al, 1973; Gogas and Skalkeas, 1975; Wallgren et al, 1977; Rosen et al, 1984; Backhouse et al, 1987; Barchielli et al, 1994). The results of this study using a large patient cohort with long-term follow-up have shown a significant difference in metastasis disease-free survival and actuarial survival between young breast cancer patients and two older age groups with primary operable invasive breast cancer. This is entirely explained by a significantly higher proportion of young patients having biological tumour characteristics, such as high grade and vascular invasion, which are associated with a worse prognosis (Elston and Ellis, 1991; Pinder et al, 1994). This results in more young patients lying within the poor prognostic group. No differences were seen when survival between age groups was compared according to the three Nottingham prognostic groups. Survival in young breast cancer patients was exactly as predicted by their prognostic index. This was confirmed by the results of the Cox multivariate analysis which failed to demonstrate young age as an independent prognostic indicator when compared to the three histological factors that comprise the NPI. The results of this study differ from those of a recent study by Bonnier et al (1995) which suggested that young age was an independent prognostic indicator after using a Cox model. Discrepancies in the results of the two studies may be explained by the small number of early-onset cases used to fit the Cox model in Bonnier's study, which may not be representative of the group as a whole. Furthermore, the Bloom and Richardson histological grading system used in that study is often criticized for being too subjective. Because histological tumour grading is a strong prognostic indicator, inconsistencies in a grading system will have a marked effect on other factors used in a multivariate analysis. The semiquantitative grading system proposed by Elston and Ellis (1991) has been shown by other centres to be reproducible with little interobserver variability (Dalton et al, 1994; Cummings et al, 1995; Frierson et al, 1995).

No differences were seen in the proportion of patients in the moderate and poor prognosis groups who received adjuvant systemic therapy according to age groups. Therefore, the effect of adjuvant systemic therapy is not expected to contribute to the differences seen in actuarial survival.

Young age alone is not an independent prognostic variable in primary breast cancer. However, adverse intrinsic biological factors are more likely to be found in breast cancers of young women.

### REFERENCES

Adami H, Malker B, Holmberg L, Persson I and Stone B (1986) The relationship between survival and age at diagnosis in breast cancer. *N Engl J Med* **315**: 559–563

Backhouse CM, Lloyd-Davies ERV, Shousha S and Burn JI (1987) Carcinoma of the breast in women aged 35 or less. *Br J Surg* **74**: 591–593

Barchielli A, Paci E, Balzi D, Geddes M, Giorgio D, Zappa M, Bianchi S and Buiatti E (1994) Population-based breast cancer survival – mammographic screening activities in central Italy. *Cancer* **74**: 3126–3134

Birks DM, Crawford GM, Ellison LG and Johnstone FRC (1973) Carcinoma of the breast in women 30 years of age or less. *Surg Gynecol Obstet* **137**: 21–25

Bonnier P, Romain S, Charpin C, Lejeune Christiane, Tubiana N, Martin P and Piana L (1995) Age as a prognostic factor in breast cancer: relationship to pathological and biological features. *Int J Cancer* **62**: 138–144

Brightmore TGJ, Greening WP and Hamlin I (1970) An analysis of clinical and histopathological features on 101 cases of carcinoma of the breast in women under 35 years of age. *Br J Cancer* **24**: 644–669

Claus EB, Risch N, Thompson WD and Carter D (1993) Relationship between breast histopathology and family history of breast cancer. *Cancer* **71**: 147–153

Cox DR (1972) Regression models and life tables. *J R Stat Soc B* **34**: 187–220

Cummings MC, Wright RG, Furnival CM, Bain CJ and Siskind V (1995) The feasability of retrospective grading of breast cancer histology slides derived from multiple pathology services. *Breast* **4**: 179–182

Dalton LW, Page DL and Dupont WD (1995) Histological grading of breast cancer: a retrospective study. *Cancer* **73**: 2765–2770

De La Rochefordiere A, Assalein B, Campana F, Scholl SM, Fenton J, Vilcoq JR, Durand JC, Poulliart P, Magdelenat H and Fourquet A (1993) Age as a prognostic factor in premenopausal breast carcinoma. *Lancet* **341**: 1039–1043

Du Toit RS, Locker AP, Ellis IO, Elston CW and Blamey RW (1990) Evaluation of the prognostic value of triple node biopsy in early breast cancer. *Br J Surg* **77**: 163–167

Earley TK, Gallagher JQ and Chapman KE (1969) Carcinoma of the breast in women under thirty years of age. *Am J Surg* **118**: 832–834

Ellis IO, Galea M, Broughton, Locker A, Blamey RW and Elston CW (1992) Pathological prognostic factors in breast cancer. II. Histological type. Relationship with survival in a large study with long-term follow-up. *Histopathology* **20**: 479–489

Elston CW and Ellis IO (1991) Pathological prognostic factors in breast cancer. I. The value of histological grade in breast cancer: experience from a large study with long-term follow-up. *Histopathology* **19**: 403–410

Forrest APM (1987) *Breast Cancer Screening*. Report to the Health Ministers of England, Wales, Scotland and Northern Ireland by a Working Group chaired by Sir Patrick Forrest. HMSO: UK

Frierson HW, Wolber RA, Berean KW, Franquemont DW, Gaffey MJ, Boyd JC and Wilbur DC (1995) Interobserver reproducibility of the Nottingham modification of the Bloom and Richardson grading system for infiltrating ductal carcinoma. *Am J Clin Pathol* **105**: 195–199

Galea MH, Blamey RW, Elston CW and Ellis IO (1992) The Nottingham prognostic index in primary breast cancer. *Breast Cancer Res Treat* **22**: 207–219

Gogas J and Skalkeas G (1975) Prognosis of mammary carcinoma in young women. *Surgery* **78**(3): 339–342

Haybittle JL, Blamey RW, Elston CW, Johnson J, Doyle PJ, Campbell FC, Nicholson RI and Griffiths K (1982) A prognostic index in primary breast cancer. *Br J Cancer* **45**: 361–366

Host H and Lund E (1986) Age as a prognostic factor in breast cancer. *Cancer* **57**: 2217–2221

Locker AP, Ellis IO, Morgan DAL, Elston CW, Mitchell A and Blamey RW (1989) Factors influencing local recurrence after excision and radiotherapy for breast cancer. *Br J Surg* **76**: 890–894

Mantel N (1966) Evaluation of survival data and two new rank order statistics arising in its consideration. *Cancer Chemother Rep* **50**: 163

Marcus JN, Watson P, Page DL, Narod SA, Lenoir GA, Tonin P, Linder-Stevenson L, Salerno G, Conway TA and Lynch HT (1996) Hereditary breast cancer – pathobiology, prognosis and BRCA1 and BRCA2 linkage. *Cancer* **77**: 697–709

Mueller CB, Ames F and Anderson GD (1978) Breast cancer in 3558 women: age as a significant determinant in the rate of dying and causes of death. *Surgery* **83**(2): 123–132

Noyes RD, Spanes WJ and Montague ED (1982) Breast cancer in women aged 30 and under. *Cancer* **49**: 1302–1307

Page DL and Anderson TJ (1987) *Diagnostic Histopathology of the Breast*. Churchill Livingstone: Edinburgh

Pinder SE, Ellis IO, Galea M, O'Rourke, Blamey RW and Elston CW (1994) Pathological prognostic factors in breast cancer. III. Vascular invasion: relationship with recurrence and survival in a large study with long-term follow-up. *Histopathology* **24**: 41–47

Remvikos Y, Magdelenat H and Dutrillaux B (1995) Genetic evolution of breast cancers. III: Age-dependent variations in the correlations between biological indicators of prognosis. *Breast Cancer Res Treat* **34**: 25–33

Ridolfi RL, Rosen PP, Port A, Kinne D and Mike V (1977) Medullary carcinoma of the breast – a clinicopathological study with ten year follow-up. *Cancer* **40**: 1365–1385

Ries LAC, Hankey BF, Miller B, Hartman AM and Edwards BK (eds) (1991) *Cancer Statistics Review 1973–1988*. NIH report no. 91-2789. National Cancer Institute: Bethesda

Rosen PP, Lesser ML, Kinne DW and Beattie EJ (1984) Breast carcinoma in women 35 years of age or younger. *Ann Surg* **9**: 191–199

Rosen PP, Lesser ML and Kinne DW (1985) Breast cancer at the extremes of age: a comparison of patients younger than 35 years and older than 75 years. *J Surg*

 © Cancer Research Campaign 1997

*Oncol* **28**: 90–96
Sant M, Gatta G, Micheli A, Verdecchia A, Capocaccia R, Crosignani P and Berrino F (1991) Survival and age at diagnosis of breast cancer in a population based cancer registry. *Eur J Cancer* **27**: 981–984
Wallgren A, Silfverswald C and Hultborn A (1977) Carcinoma of the breast in women under 30 years of age. *Cancer* **40**: 916–923

© Cancer Research Campaign 1997